# The Overnight Retention of Novel Metaphors Associates With Slow Oscillation–Spindle Coupling but Not With Respiratory Phase at Encoding

**DOI:** 10.3389/fnbeh.2021.712774

**Published:** 2021-08-31

**Authors:** Risto Halonen, Liisa Kuula, Minea Antila, Anu-Katriina Pesonen

**Affiliations:** Sleepwell Research Program, Faculty of Medicine, University of Helsinki, Helsinki, Finland

**Keywords:** metaphor, respiration, sleep spindle, slow oscillation, memory, phase lag index

## Abstract

Accumulating evidence emphasizes the relevance of oscillatory synchrony in memory consolidation during sleep. Sleep spindles promote memory retention, especially when occurring in the depolarized upstate of slow oscillation (SO). A less studied topic is the inter-spindle synchrony, i.e. the temporal overlap and phasic coherence between spindles perceived in different electroencephalography channels. In this study, we examined how synchrony between SOs and spindles, as well as between simultaneous spindles, is associated with the retention of novel verbal metaphors. Moreover, we combined the encoding of the metaphors with respiratory phase (inhalation/exhalation) with the aim of modulating the strength of memorized items, as previous studies have shown that inhalation entrains neural activity, thereby benefiting memory in a waking condition. In the current study, 27 young adults underwent a two-night mixed-design study with a 12-h delayed memory task during both sleep and waking conditions. As expected, we found better retention over the delay containing sleep, and this outcome was strongly associated with the timing of SO–spindle coupling. However, no associations were observed regarding inter-spindle synchrony or respiratory phase. These findings contribute to a better understanding of the importance of SO–spindle coupling for memory. In contrast, the observed lack of association with inter-spindle synchrony may emphasize the local nature of spindle-related plasticity.

## Introduction

The benefits of sleep for memory retention have been widely acknowledged. Sleep protects memory function from wake–time interference and actively consolidates labile traces (Rasch and Born, 2013). Sleep spindles are considered biomarkers of sleep-related memory processing, promoting hippocampal–neocortical dialog (Ngo et al., 2020) and synaptic calcium-dependent plasticity processes (Peyrache and Seibt, 2020). Accordingly, studies have frequently reported that the amount of spindles is associated with better memory outcomes after delay containing sleep (Gais et al., 2002; Clemens et al., 2005; Halonen et al., 2019). Sleep spindles do not occur in isolation. Recent research has stressed the importance of coordinated activity between cortical slow oscillations (SOs), sleep spindles, and hippocampal sharp-wave ripples with respect to facilitating active memory consolidation (Klinzing et al., 2019). During SOs, the synchronized depolarization of large neuronal ensembles biases synapses toward potentiation (Chauvette et al., 2012). Concomitantly, depolarization reaching the thalamic reticular nucleus triggers a spindle event that travels back to the cortex (Steriade, 2006). Spindles mediate hippocampal–neocortical coupling, which may be further enhanced by spindle-nested hippocampal sharp-wave ripples (Ngo et al., 2020). This interplay provides a powerful window for memory consolidation, especially when the spindle occurs at the SO upstate (Klinzing et al., 2019). Behavioral studies in humans have shown that the accuracy of SO–spindle coordination in the fronto-central brain areas is associated with declarative learning (Helfrich et al., 2018; Mikutta et al., 2019; Muehlroth et al., 2019; Hahn et al., 2020).

The dynamics of temporally overlapping spindles in separate cortical locations is an understudied phenomenon in memory research. The relatively frequent simultaneous or nearly simultaneous occurrence of spindles in more than one electrode location is a consistent finding across studies (O’Reilly and Nielsen, 2014; Frauscher et al., 2015; Souza et al., 2016; Piantoni et al., 2017) and is proposed to reflect a “matrix” (widespread) pathway with respect to sleep spindles (Bonjean et al., 2012; Piantoni et al., 2016). Differing from “core” pathways in terms of involved thalamic nuclei and projected cortical layers, the matrix neurons are implicated in widespread, synchronized activity (in contrast to locally occurring spindles) (Piantoni et al., 2016). A number of experimental studies have examined the dynamics of propagated/global spindles (e.g., the velocity and preferred direction of spindle propagation) (O’Reilly and Nielsen, 2014; Frauscher et al., 2015; Souza et al., 2016; Piantoni et al., 2017). While these findings are rather ungeneralizable due to divergent methods, one articulate finding is the rapid phase synchronization between overlapping spindles (Souza et al., 2016). Coherent neural firing between distinct brain areas is considered to mark efficient communication between neuronal groups (Fell and Axmacher, 2011) and is associated with working memory performance (Schack and Weiss, 2005; Palva et al., 2010), executive function (Mizuhara and Yamaguchi, 2007; Sadaghiani et al., 2012; Cavanagh and Frank, 2014), and delayed recognition memory (Rutishauser et al., 2010). However, it remains unclear whether the synchronization properties of propagated spindles have specific implications for long-term memory retention beyond a hypothetical role (Piantoni et al., 2016).

Respiration can also influence memory processing. For example, one study (Arshamian et al., 2018) found better odor recognition when nasal respiration was maintained over the retention period (compared with oral airflow). The authors speculated a possible mechanism for increased sharp-wave ripples during inhalation based on findings among rodents (Liu et al., 2017). Another study (Zelano et al., 2016) examined how the phase of respiration modulates memory in humans. They found the most accurate picture recognition for items that were encoded and retrieved during nasal inhalation, as compared with exhalation and/or oral airflow. Concordantly, intracranial electroencephalography (EEG) showed inhalation-tied elevations in oscillatory power and synchrony (including the theta range) in structures involved in mnemonic processes, such as the hippocampus and amygdala (Zelano et al., 2016). These findings suggest that respiratory phase could interact with sleep-dependent memory consolidation. Notably, the levels of limbic facilitation (Goldstein and Walker, 2014; Hermans et al., 2014) and theta activity (Benchenane et al., 2010; Cohen et al., 2015) during encoding are considered to indicate the *saliency* of newly potentiated memory traces. A line of research proposes that salient memories are preferably strengthened during sleep (Peyrache et al., 2009; Benchenane et al., 2010; Bennion et al., 2016; Alger et al., 2019), and sleep spindles are possibly involved in this process (Studte et al., 2017; Alger et al., 2019). Even though the case for selectiveness in memory consolidation during sleep lacks decisive evidence (Davidson et al., 2020), it is compelling to ask whether the respiratory phase that modulates theta coherence would then affect the “priority” of newly potentiated memory traces through active consolidation mechanisms. However, to our knowledge, no study thus far has examined the consequences of respiratory phase on memory during a sleep-filled delay.

Studies on verbal memory and sleep spindles and/or SO coupling have commonly deployed word pair or word list tasks (Mednick et al., 2013; Mikutta et al., 2019; Hahn et al., 2020). However, a yet-unstudied form of verbal material in sleep-related memory retention is metaphoric association. According to evidence from imaging studies, the processing of metaphors correlates with more widespread neural activation as compared with processing non-metaphors (Bambini et al., 2011), whereas novelty in linguistic associations induces bilateral recruitment (in contrast to left-lateralized familiar associations; Arzouan et al., 2007; Mashal and Faust, 2009; Bohrn et al., 2012; Lai et al., 2015; Diaz and Eppes, 2018). The current state-of-the-art literature in sleep-related memory consolidation is lacking an understanding of how these more complex memory representations are processed during sleep.

In this study, we investigated how verbal memory retention is reflected by (1) known sleep-dependent consolidation mechanisms (i.e., SO–spindle coupling) and (2) inter-spindle synchrony, an unstudied potential indicator of widespread memory processing. Our protocol contained delayed retention over both sleep and wake conditions in order to assess if retention (and its association with sleep-related mechanisms) showed differences between these conditions. In addition, motivated by research on respiration and memory, we devised an experimental setting integrating paced breathing into memory tasks in order to examine if encoding-tied respiratory phase affects retention performance. The material in the memory task consisted of novel metaphoric associations (Herkman and Service, 2008). Our main hypothesis was that we would observe a statistically significant positive association between memory retention over sleep conditions and the timing of SO–spindle coupling (i.e., the upstate-preference of spindles). We examined this phenomenon in non-rapid eye movement (NREM; N2 and N3 combined) sleep as well as separately for N2 and N3 stages; these stages potentially differ in coupling dynamics (Cox et al., 2018). We also expected to find improved recall for metaphors encoded during inhalation, as compared with exhalation.

## Materials and Methods

### Participants

**FIGURE 1:**
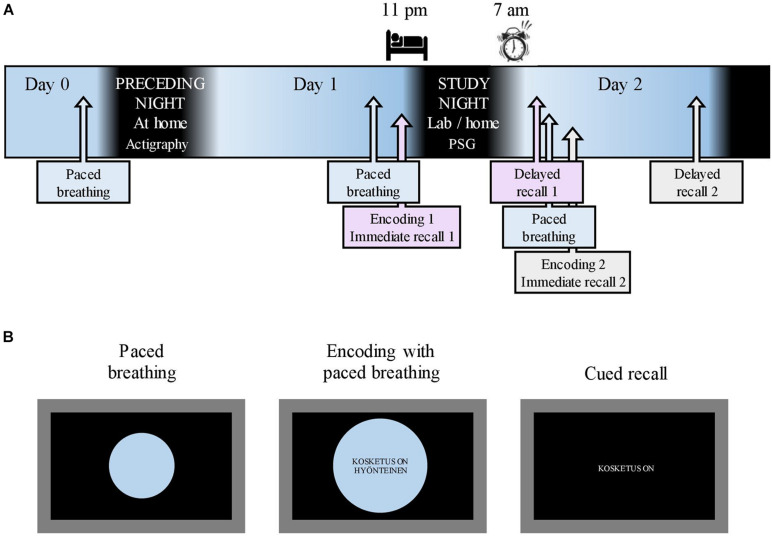
Study flow and memory tasks. **(A)** The study flow starting after the acquisition of actigraphy on Day 0. A 10-min breathing exercise, paced with an expanding/shrinking circle, was conducted on the evening of Day 0 as well as before each encoding. Respiratory phase-tied encoding of novel metaphors was followed by immediate recall on Day 1 evening and Day 2 morning. Delayed recall took place in the morning and evening of Day 2. Sleep opportunity during the study night was between 11:00 p.m. and 07:00 a.m. **(B)** A 10-min breathing exercise was paced with a shrinking/expanding circle on a laptop screen. During encoding, the participants maintained paced breathing while novel metaphors were displayed in the center of the screen. Cued recall (immediate and delayed) was done without the breathing circle. English translation for the example metaphor: “(A) touch is (an) insect” in the middle screen represents encoding and “(A) touch is” in the right screen represents recall.

The initial sample consisted of 29 young adults (23 females) living in the capital area of Finland. The participants were recruited *via* different channels; 11 were invited from the previously studied *SleepHelsinki!* cohort (see details of the cohort^1^), 15 were students at the University of Helsinki who were contacted *via* e-mail lists and social media channels within student societies, and three participants were invited through personal contacts. All participants were offered 100 € monetary compensation for their participation. Measurements were performed between June 2020 and January 2021. We screened the participants for learning difficulties and excluded one participant due to a reading disability. We also administered questionnaires for depressive symptoms [Beck Depression Inventory (BDI)] (Beck et al., 1996) and generalized anxiety symptoms [Generalized Anxiety Disorder-7 (GAD-7)] (Williams, 2014) in order to address this symptomology in analyses evaluating memory function. Due to technical issues, we lost Day 2 (Figure 1A) delayed recall data from two participants, one of whom had missing immediate recall data.

Written informed consent was obtained from all the participants prior to study participation. The study was approved by the Helsinki University Hospital Ethics Committee, and all components of the study were conducted in accordance with the Declaration of Helsinki and its later amendments.

### Study Design

We deployed a mixed-design study examining memory outcomes in a within-subjects manner with respect to the delay condition (for sleep and wake conditions) and the respiratory phase (inhalation/exhalation). The associations between memory outcomes and sleep oscillation measures were compared between the participants.

After enrolling in the study, the participants completed electronic questionnaires concerning background data (BDI, GAD-7, learning impairments, and health status). On Day 0 (Figure 1A), the participants retrieved the actigraphy from the laboratory. That same evening, research assistants contacted the participants *via* Zoom to administer a 10-min nasal breathing exercise, paced by an expanding and shrinking circle on a shared laptop screen (Figure 1B; see section “Respiratory Phase” for details concerning respiration). All participants slept at home on the night between Day 0 and Day 1. To mitigate any reluctance to take part in the study during the ongoing COVID-19 pandemic, the participants were allowed to choose whether the study night (i.e., between Day 1 and 2) measurements were conducted in the sleep laboratory or at the participant’s home. Fifteen participants opted for laboratory measurements. On the evening of Day 1 (mean time, 08:01 p.m.; SD, 11 min), the research assistant met the participants at their homes or at the sleep laboratory. After a paced breathing exercise, participants underwent the first metaphor encoding maintaining paced breathing (Figure 1B; see section “Material and Memory Task” for details on the memory task). Ten minutes later, an immediate cued recall was administered (Figure 1B). A polysomnography (PSG) device was attached; the participants had sleep opportunities between 11:00 p.m. (mean bedtime, 11:25 p.m.; SD, 34 min) and 07:00 a.m. (mean awakening, 06:59 a.m.; SD, 4 min). The first delayed recall was performed the next morning (mean, 07:43 p.m.; SD, 16 min). Shortly after, a new paced breathing exercise was performed, followed by the second metaphor encoding and immediate recall. The participants spent Day 2 of the study performing their normal activities. The final delayed recall took place in the evening (mean, 08:02 p.m.; SD, 2 min) on Day 2.

### Material and Memory Task

The memory task was run with Neurobehavioral Systems Presentation software (version 22.0; Neurobehavioral Systems, Inc., Berkeley, CA, United States). The task material consisted of two sets of 48 metaphors: one for overnight memory retention and one for over day memory retention. Additionally, each set was divided into subsets of 16 and 32 metaphors as well as immediate and long-delay recall, respectively. The metaphors were chosen based on work on psycholinguistic dimensions (Herkman and Service, 2008), where participants used a scale of 1–7 to evaluate non-conventional metaphors in nine dimensions, including ease (how easy a metaphor was to envision) and liveliness (how vivid and detailed the metaphor was). To eliminate bias in memory performance due to differing metaphor difficulty, we matched the two sets in terms of the ease dimension (*t*-test *p* = 0.929), with ranges of normative ease of 2.38–5.84 and 2.52–5.90 in the two sets. The presentation order of these sets was counterbalanced. We also balanced subsets of immediate and delayed recall (*p* = 0.958). For our analyses, we inverted the ease values to represent metaphor difficulty. As an example, the metaphor “(A) malicious remark is a bullet” has lower normative difficulty (2.98) than the metaphor “(A) clever joke is (a) splint” (5.36). The parentheses in the example metaphors denote that there are no articles in Finnish.

On both occasions of encoding, the participants were shown 48 written metaphors on a laptop screen, with instructions to form a mental image of the metaphors. Each metaphor was shown for 4.25 s within each phase of respiration (see section “Respiratory Phase”), with a 1.5-s interval until the next metaphor was shown. The participants underwent three successive encoding rounds, after which the metaphors were no longer displayed (e.g., during immediate recall when providing an incorrect answer). Thus, the retention and forgetting between immediate and delayed recall could be reliably examined.

The delay from encoding to immediate recall was 10 min (16 metaphors), and delayed recall was 12 h (32 metaphors). In the recall, the participants were shown the beginning of a metaphor on a laptop screen [(e.g., “(A) touch is (an)”] in random order and were asked to type the missing (last) word (e.g., “*insect*”). Responses were scored so that 1 point was given for a correct word (the plural form was counted as correct), and 0.5 points were given if the response was a synonym (e.g., “bug”) or a higher/lower abstraction of the correct word (e.g., “mosquito”). The responses were first scored by two researchers independently and then merged into a combined scale based on mutual agreement on ambiguous responses. The memory retention outcome was calculated so that the percentage of correct responses in delayed recall was subtracted from the percentage of correct responses in immediate recall, resulting in change scores (ΔSleep and ΔWake) corresponding to the delayed condition.

### Respiratory Phase

The respiratory phase was included in the memory task in the following manner. In the evening of study Day 1 and before each encoding, the participants were administered a nasal breathing exercise for 10 min to familiarize them with the breathing procedure. Breathing was paced with a circle shown on a laptop screen (Figure 1B); the participants were instructed to inhale while the circle expanded and to exhale while it shrank. One breathing cycle lasted for 11.50 s in total. Toward the end of a phase, the expanding/shrinking slowed down, with a 1-s halt at the phase peak. The exact timing was piloted, with eight people representing the age group of the study sample for the purpose of approximating natural, relaxed nasal breathing and minimizing distraction from encoding. During encoding, metaphors were presented to the participants in the center of the breathing circle (Figure 1B); one metaphor was presented during each respiratory phase. All the participants underwent three successive encoding rounds. Specific metaphors were presented at the same respiratory phase in each round.

Respiration-related memory outcomes included pooled immediate recall (that is, the combined score of correct responses across both immediate recall sessions; Day 1 evening and Day 2 morning) for both inhale-phased and exhale-phased metaphors. Phase-specific retention scores for both delay conditions were calculated separately for inhale- and exhale-phased metaphors by subtracting the delayed recall percentage from the immediate recall percentage.

### Actigraphy

All participants wore Philips Actiwatch 2 actigraphs to screen for highly deviant sleep durations before the laboratory night. The actigraphs were worn for 2 days, between the evening on Day 0 and the evening on Day 2 (Figure 1A).

### Polysomnography Protocol and Preprocessing

All recordings were performed using either SOMNOscreen plus or SOMNOscreen HD (SOMNOmedics GmbH, Randersacker, Germany). The trained research nurse attached gold cup electrodes at six EEG locations [frontal (F) hemispheres F3 and F4; central (C) C3 and C4; occipital (O) O1 and O2; mastoid (A1, A2)]. Electrooculograms (EOGs) and chin electromyograms (EMGs) were measured using disposable adhesive electrodes (Ambu Neuroline 715; Ambu A/S, Ballerup, Denmark), with two locations for EOG and three locations for EMG. An online reference (Cz) and a ground electrode in the forehead were used in the current study. The sampling rate was 256 Hz (the hardware filters for SOMNOscreen plus were 0.2–35 Hz). PSG data were scored manually using the DOMINO program (v2.7; SOMNOmedics GmbH, Germany) in 30-s epochs into N1, N2, N3 (SWS), REM, and wake according to AASM guidelines (AASM Manual for the Scoring of Sleep and Associated Events, 2007). Movement arousals were also marked.

The manually scored PSG signals were converted to EDF format in DOMINO software (SOMNOmedics GmbH, Germany) and then further analyzed using the functions of EEGlab 14.1.2b (Delorme and Makeig, 2004) running on MATLAB R2018a (MathWorks, Inc., Natick, MA, United States). All signals were digitally band-passed and filtered offline from 0.2 to 35 Hz (with a Hamming windowed sinc zero-phase FIR filter; cutoff, −6 dB), at 0.1 and 35.1 Hz, respectively, and re-referenced to the average signal of A1 and A2 electrodes. Electrodes located at F3, F4, C3, and C4 were included in further analyses; all further analyses were conducted on sleep epochs that were scored as N2 or N3.

### Sleep Spindle Detection

The preprocessed EEG data were further band-pass filtered (order 2816) in the 12- to 16-Hz frequency band. From the filtered signal, spindles were extracted using a method based on an automated detection algorithm described by Ferrarelli et al. (2007). The threshold values for finding the spindle peak amplitude in each channel were defined by the mean of the channel amplitude (μV) multiplied by 5. The putative spindle’s amplitude was required to stay over the mean channel amplitude multiplied by 2 for 250 ms in both directions from the peak maximum, resulting in minimum spindle duration of 0.5 s. Thus, we used channel-wise threshold definitions, considering that signals may vary across channels and between individuals. The maximum cutoff for spindle length was set to 3.0 s, and the maximum peak amplitude was set to 200 μV. In addition, the signal amplitude between spindles was required to stay under the lower threshold for 78.1 ms, which is approximately the duration of one period of sine at 13 Hz; this requirement was implemented in order to prevent false alarms. Finally, we excluded spindle-like bursts that occurred during arousal. We ran the detection for NREM sleep (N2 + N3) epochs and separately for N2 and N3 epochs.

### Slow Oscillation Detection

Slow oscillations were detected with an adapted algorithm developed by Ngo et al. (2015) using the Wonambi EEG analysis toolbox (Piantoni and O’Byrne, 2021; Wonambi: EEG analysis toolbox v.6.13^2^). The signal was first low-pass filtered at 3.5 Hz. All negative and positive amplitude peaks were identified between consecutive positive-to-negative zero-crossings, comprising a full phase cycle. Zero-crossing intervals within the duration of 0.8–5 s were included, corresponding to the 0.2–1.25 frequency range. Finally, mean values for positive and negative peak potentials were calculated, and these events were denoted as SOs where the negative peak was lower than the mean negative peak and where the positive-to-negative peak amplitude difference exceeded the mean amplitude difference. We ran the detection procedure separately for NREM, N2, and N3 sleep.

### Phase-Amplitude Coupling

We operationalized phase-amplitude coupling (PAC) as a modulation index (MI), estimated with an adaptation of the Kullback–Leibler distance proposed by Tort et al. (2010) with methods from Tensorpac (Combrisson et al., 2020). We calculated channel-wise MIs from all 30-s N2 and N3 sleep epochs with SO range (0.2–1.25) as the phase frequency and spindle range (12–16 Hz) as the amplitude frequency range. The phase was first divided into 18 bins, each at 20°. The mean spindle range amplitude was then computed for each bin. Finally, the empirical probability distribution was obtained by dividing the measured amplitude inside each bin by the sum of the bins. MI represents the difference between this distribution and the uniform distribution. To correct the PAC measure for noise, we calculated the surrogate distribution by splitting the amplitude blocks at a random time point, swapping them, and calculating a PAC measure with the original phase data (Bahramisharif et al., 2013). The distribution of surrogate values was obtained by repeating this procedure 1,000 times. Finally, a corrected PAC was calculated by subtracting the surrogate means from the uncorrected MIs and then dividing by the surrogate standard deviation.

### Preferred Phase

Separately for NREM, N2, and N3 sleep, we calculated the preferred phase (PP) of spindles within SOs (that is, the phase angle of the SOs at which the peak spindle amplitudes aligned). We examined this coupling within each EEG channel. First, we identified spindles where the amplitude peaked within an SO cycle (i.e., SO–spindles). Next, we band-pass filtered the EEG signal to 0.2–1.25 Hz, Hilbert-transformed the SO signal, and extracted the instantaneous phase at the SO–spindle peaks. The examined variables were SO–spindle% (i.e., the percentage of spindles occurring during SO, out of all spindles), the circular mean angle in degrees, and the percentage of spindle peaks (out of all SO–spindle peaks) occurring at the upstate point (± 45° from 0) for each participant.

### Inter-Spindle Synchrony

**FIGURE 2:**
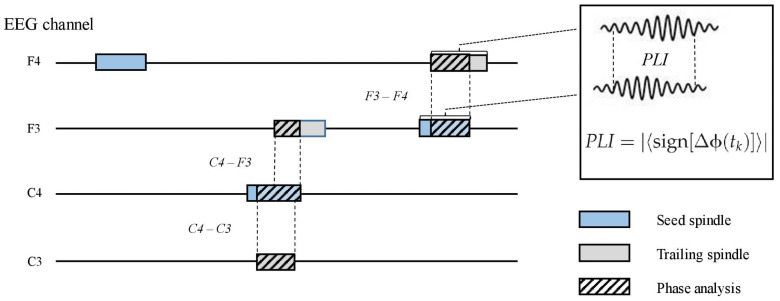
A schematic picture of parametrizing inter-spindle synchrony. Seed spindles (light blue) may be overlapped by one or more spindles in other electroencephalography channels (trailing spindles, light gray). Phase lag index (PLI) between the seed and trailing spindles was calculated (striped pattern) if the overlap was equal or higher than 0.3 s. Sign, *signum* function; ΔΦ, phase difference; t_*k*_, samples across the overlapping signal.

To measure inter-spindle synchrony in separate channels, we first identified co-occurring spindles between the F3, F4, C3, and C4 channels. This was done offline in the following steps. (1) Proceeding chronologically, when a spindle occurred after a spindle-free period in any channel, we examined if another spindle overlapped (i.e., peaked within the duration of the first spindle) at any other channel. (2) In the case of an overlapping spindle, we examined which spindle peaked first and nominated it as *the seed* spindle. (3) Any co-occurring spindle was designated as *a trailing* spindle. Thus, a seed spindle could occur alone or be overlapped by one, two, or three (different channel) trailing spindles. The direction of the overlap (i.e., seed to trailing; e.g., F3–C3) was recorded. For each electrode pair (seed–trailing), we calculated the propagation latency, overlap duration, and propagation density (PD), which represent the probability of a certain pair (e.g., F3–C3) occurring among all seed spindle events in the channel (e.g., F3) (Figure 2). To include only those spindle pairs with an overlap delay plausibly underlain by wave propagation, we excluded those pairs in which the delay between the peaks was below 8 ms. The selected delay was based on a previous study on spindle propagation (O’Reilly and Nielsen, 2014).

The phase lag index (PLI) (Stam et al., 2007) was computed for all seed–trailing pairs that overlapped by more than 0.3 s (Figure 2). For the pairs of overlapping band-pass-filtered EEG signals, we first extracted the phase angle in radians for each sample after the Hilbert transform. The asymmetry of phase differences over the signal pairs was obtained with the *signum* function, resulting in pair-specific raw PLIs ranging from 0 (random phase difference) to 1 (fixed phase difference). Again, we included spindle pairs that did not overlap instantly (<8 ms). The obtained raw PLIs were further contrasted with randomized spindle-PLIs to highlight the synchrony between overlapping spindles, in contrast to non-overlapping spindles. To this end, the contrast spindle-PLIs were created by calculating the PLI values between each seed spindle and a randomly chosen spindle event from the same participant; this procedure was repeated 100 times. Channel-pair-wise corrected PLIs (cPLIs) were obtained by subtracting the contrast PLIs from the raw PLIs. The cPLIs were categorized according to the direction (e.g., F3–C3) for each participant.

For memory outcome analyses, we averaged both PD and cPLI values into frontal to central (F–C), central to frontal (C–F), left to right (L–R), and right to left (R–L) means. We only averaged non-diagonal electrode pairs (for example, F-to-C, including F3–C3 and F4–C4, and not F3–C4 or F4–C3).

### Statistical Analyses

In the analyses evaluating metaphor difficulty level, one metaphor was considered an observation unit. The association between recall probability in immediate recall and metaphor difficulty was tested using one-way analysis of variance (ANOVA). For delayed recall, we constructed a mixed ANOVA model with recall probability (for both sleep and wake conditions) as dependent variables and constructed metaphor difficulty as a continuous independent variable in order to examine the main effects of metaphor difficulty as well as its interactions with delay conditions.

One-way ANOVA was used to test whether the place of measurement was related to sample characteristics (i.e., age, sleep measures, questionnaire scores, and recall results) or sex-induced differences in dependent and independent variables. We used repeated-measures ANOVA to test if (1) immediate recall scores differed between evening and morning recalls, (2) if the delay condition affected the delayed retention outcome (ΔSleep and ΔWake), and (3) if the respiratory phase affected pooled immediate recall. The effect of respiratory phase across the delay conditions was tested with a mixed ANOVA model, assigning two levels for phase (inhale and exhale) and two levels for the delay condition (sleep and wake) as within-subject variables. The associations between memory retention (ΔSleep and ΔWake), SO–spindle coupling variables (MI, SO–spindle%, and Upstate%), and inter-spindle variables (PD and cPLI) were tested using linear regression analyses. A quadratic regression was used to examine the association between memory retention and PP_*Mean*_ because this variable represents values (degrees) distributed on a circular (not linear) plane.

Rayleigh’s test of non-uniformity was used to test the circular distribution of PP_*Mean*_ values at the group level. We compared the frontal and central NREM PP_*Mean*_ distributions using the Watson–Williams test. The SO–spindle coupling variables in N3 and N2 sleep were compared using a pairwise *t*-test. The association between PD and cPLI grand mean was tested using Pearson’s correlation. We tested for statistical differences between the variable means using Friedman’s test, and this test was conducted separately for PD and cPLI.

Sex was partialed out from the memory outcome variables in all analyses. In order to evaluate how possible confounders influenced the statistical significance of the results, we also constructed a model controlling for sleep duration, age, BDI score, and GAD-7 score (that is, the control model).

The nominal level of statistical significance was set at *p* < 0.05. Statistical analyses were performed using IBM SPSS Statistics for Windows, version 27.0 (IBM Corp., Armonk, NY, United States) or the CircStat Toolbox for Matlab R2018a (Berens, 2009).

## Results

### Sample Characteristics

Table 1 presents sample characteristics, including age, sleep measures, questionnaire scores, and raw memory task scores. We also compared these characteristics between home-measured and laboratory-measured participants, demonstrating higher sleep durations during the previous night and the study night (*p* = 0.021 and 0.027, respectively) as well as higher N3 percentage (*p* = 0.012) for those who slept at home.

**TABLE 1:** Sample characteristics.

		Range	Mean	SD	*P*
Age		19–41	22	4.3	0.173
Sleep duration, previous night (h:mm)		5:50–8:37	7:10	0:46	0.021*
Sleep duration, study night (h:mm)		4:18–7:49	6:38	0:50	0.027*
	N1%	1.4–17.26	5.9	3.6	0.114
	N2%	21.6–49.3	35.5	8.1	0.555
	N3%	8.2–41.4	24.6	7.4	0.012*
	REM%	7.9–27.9	18.2	5.1	0.274
WASO (h:mm)		0:05–2:07	0:29	0:32	0.980
BDI score		0–31	10.9	9.3	0.840
GAD-7 score		1–15	5.4	4.0	0.319
Immediate recall, evening		9.5–16	13.2	2.0	0.444
Immediate recall, morning		10–16	13.8	1.7	0.918
Delayed recall, sleep		10–31.5	25.0	6.0	0.537
Delayed recall, wake		2.5–26	17.2	5.8	0.475

SD, standard deviation; *p*, *p*-value of the difference between measurement place (home/laboratory); N1–3, non-rapid eye movement sleep stages 1–3; REM, rapid eye movement sleep; WASO, wake after sleep onset; BDI, Beck Depression Inventory; GAD-7, Generalized Anxiety Disorder-7 questionnaire.

*p-value < 0.05.

Exploring the dependent and independent variables for any differences related to sex and measurement place (home/laboratory) revealed better retention over sleep in females (i.e., ΔSleep; *F* = 6.508, *p* = 0.017). When evaluating the distributions, we found that one participant showed extremely high values for three SO–spindle coupling variables (>3 SD). This participant was excluded from further analyses.

### Metaphor Difficulty

**FIGURE 3:**
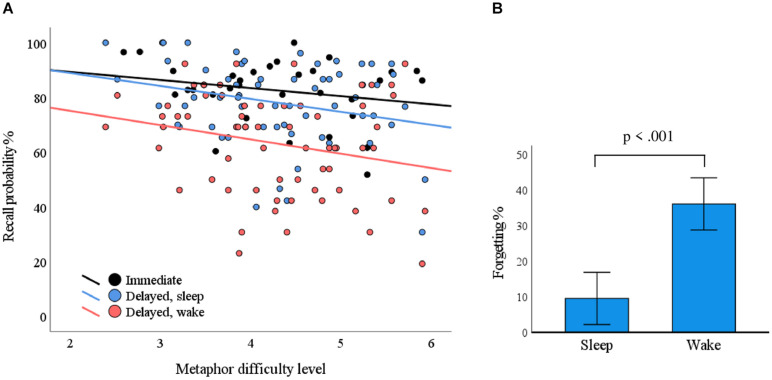
The impact of metaphor difficulty and delay conditions on recall performance. **(A)** Each dot represents a metaphor. The probability for correct responses depended on the metaphor difficulty level in the delayed condition (*p* = 0.023; blue line, sleep; red line, wake). **(B)** The delay over sleep associated with less forgetting as compared with the wake condition (*p* < 0.001).

The mean (standard deviation) recall probabilities for overnight, daytime, and immediate recall conditions were 78.1% (15.1%), 63.1% (18.6%), and 82.5% (11.5%), respectively. The metaphor difficulty level was associated with recall probability in delayed recall (*F* = 5.403, *p* = 0.023) but not in immediate recall (*F* = 1.613, *p* = 0.214). The delay condition (sleep/wake) did not interact with difficulty level in terms of recall probability (*F* = 0.046, *p* = 0.831); that is, the condition did not cause differences in associations between difficulty level and recall probability. Recall probability as a function of metaphor difficulty level is plotted in Figure 3A.

### Sleep, Wake, and Breathing Phase

Within-subject ANOVA of immediate recall performance showed no statistically significant differences between the evening and morning (*F* = 0.666, *p* = 0.433). We used a mixed ANOVA with ΔSleep and ΔWake as within-subject variables to test if metaphor retention differed over sleep conditions vs. waking conditions. We found that retention was statistically significantly better over sleep conditions (*F* = 39.743, *p* < 0.001; Figure 3B). Breathing phase did not have a statistically significant influence on pooled immediate recall (*F* = 0.075, *p* = 0.787) or delayed recall (*F* = 0.063, *p* = 0.804), nor did it interact with the delayed condition (*F* = 0.491, *p* = 0.491).

### Slow Oscillation–Spindle Coupling

**FIGURE 4:**
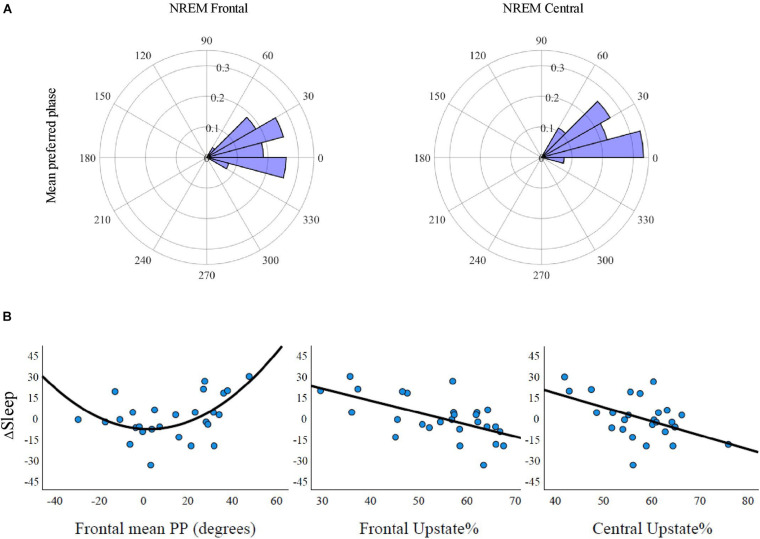
Mean preferred phase and memory outcomes. **(A)** Circular histograms of individual mean preferred phase angles of spindle peak amplitude in a slow oscillation (SO) cycle for frontal and central SO–spindle complexes. The mean angles were non-uniformly distributed in frontal (*p* < 0.001) and central (*p* < 0.001) electrodes and showed a tendency to cluster near SO upstate (i.e., 0°). There were no statistically significant differences in preferred phase between the frontal and central areas (*p* = 0.110). **(B)** The coupling accuracy between SO upstate and spindle peak amplitude in predicted overnight memory retention. ΔSleep, reflecting overnight forgetting, was associated with the frontal mean circular direction (left; *p* = 0.019) and with the frontal and central percentage of SO–tied spindles peaking at the SO upstate (± 45°) (*p* < 0.001 and *p* = 0.008, respectively).

Assessing the uniformity of PP_*Mean*_ values over the sample with Rayleigh’s test revealed highly non-uniform distributions in both frontal (*p* < 0.001) and central (*p* < 0.001) SO–spindles for NREM, N2, and N3 sleep. The grand mean degrees (and standard deviations) in NREM sleep for frontal and central SO–spindle PPs, respectively, were 12.7° (20.1°) and 20.9° (17.2°) (Table 2). Figure 4A displays the tendency of mean PP angles to cluster near the SO upstate (0°) in NREM sleep. Frontal and central PP_*Mean*_ values did not differ in NREM, N2, or N3 sleep (*p*-values ≥ 0.083). Comparison of the coupling measures between N2 and N3 sleep showed that PAC, SO–spindle%, PP_*Mean*_, and Upstate% were statistically significantly higher in N3 sleep both frontally and centrally (*p*-values ≤ 0.010) (Table 2).

**TABLE 2:** Oscillation characteristics.

	NREM		N2		N3		N2 vs. N3
	Mean	SD	Mean	SD	Mean	SD	*p*
Spindle density F	4.44	0.81	4.61	0.74	4.48	0.97	0.347
Spindle density C	4.59	0.76	4.72	0.79	4.62	0.87	0.504
Slow oscillations F	1,946	433	728	159	1,023	321	<0.001***
Slow oscillations C	1,859	390	730	175	978	290	<0.001***
SO–spindle% F	13.7	4.4	12.7	4.4	16.0	5.3	<0.001***
SO–spindle% C	12.8	4.0	11.7	4.4	15.7	5.1	<0.001***
Spindle–SO% F	17.9	5.6	27.3	8.6	15.1	5.8	<0.001***
Spindle–SO% C	17.9	5.6	25.2	7.1	16.2	5.6	<0.001***
PP_*Mean*_ F	12.7°	20.1°	19.5°	23.5°	5.6°	16.6°	00.010*
PP_*Mean*_ C	20.9°	17.2°	30.9°	20.6°	11.8°	14.9°	<0.001***
Upstate% F	54.64	10.72	46.32	10.36	56.18	9.83	<0.001***
Upstate% C	58.00	7.50	45.97	9.13	60.56	6.6	<0.001***

*Slow oscillations were summed over frontal/central electrodes.*

F, frontal; C, central; Spindle density, spindles per minute, averaged over frontal/central electrodes; SO–spindle%, the percentage of spindles occurring during slow oscillation out of all spindles; Spindle–SO%, the percentage of slow oscillations with a spindle occurrence; NREM, non-rapid eye movement sleep; N2–3, NREM sleep stages 2 and 3; *p*, *p*-value pairwise *t*-test comparing N2 and N3 sleep values. ***p-value < 0.001.

*p-value < 0.05.

### Slow Oscillation–Spindle Coupling and Novel Metaphor Learning

First, we examined if MI between the oscillation ranges of 0.2–1.25 Hz (phase frequencies) and 12–16 Hz (amplitude frequencies) was associated with the retention of metaphors over sleep and wake conditions. In both the frontal and central derivations, MI over NREM sleep (N2 and N3 combined) was not significantly associated with ΔSleep (*t* = −0.111, *p* = 0.913 and *t* = −0.129, *p* = 0.898, respectively) or with ΔWake (*t* = 0.305, *p* = 0.764 and *t* = 0.866, *p* = 0.397). No statistically significant associations were found when studying MI in N2 and N3 sleep separately (*p*-values ≥ 0.288).

We proceeded to test how the interplay between SO and spindle events is related to delayed memory outcomes. See Table 2 for details on spindle density, SO amount, and coupling probability. First, SO–spindle% was not associated with ΔSleep or ΔWake in NREM or N2 sleep (*p* ≥ 0.185). However, we found statistically significant associations with respect to N3 sleep, where the frontal SO–spindle% was statistically significant for ΔSleep (frontal: *t* = −2.085, *p* = 0.047; control model *t* = −2.476, *p* = 0.022). The central SO–spindle% in N3 was statistically significant in the control model (control model *t* = −2.624, *p* = 0.016; unadjusted model *t* = −2.027, *p* = 0.053). ΔWake was not associated with the N3 SO–spindle ratio (*p*-values ≥ 0.407).

Examining how PP over NREM sleep was associated with overnight forgetting of novel metaphors revealed a statistically significant association with frontal (*t* = 2.508, *p* = 0.019) but not central (*t* = 1.532, *p* = 0.139) PP_*mean*_ angles (Figure 4B). In addition, the percentage of SO–spindles occurring during the upstate (± 45° from 0°) was statistically significantly associated with overnight retention in frontal (*t* = −3.796, *p* < 0.001) and central (*t* = −2.880, *p* = 0.008) derivations, indicating that less forgetting occurred along with an increased probability of spindles peaking at the SO upstate. Running the analyses with the control model did not change the significance status of the results (see Supplementary Table 1 for the channel-wise regression and control model results). Investigation of PP during N2 and N3 sleep separately showed that PP_*mean*_ statistically significantly predicted ΔSleep in the central derivation (*t* = 2.372, *p* = 0.026) during N3 sleep, whereas frontal Upstate% showed statistically significant associations during both N2 (*t* = −2.809, *p* = 0.010; control model: *t* = −2.609, *p* = 0.016) and N3 sleep (*t* = −3.280, *p* = 0.003; control model: *t* = −2.616, *p* = 0.016) (Supplementary Table 2). Finally, we conducted secondary analyses controlling for whether the quantity of spindles or SOs drove the statistically significant associations between PP measures and ΔSleep. To this end, we ran again the analyses showing statistically significant associations with ΔSleep (i.e., frontal PP_*Mean*_ in NREM and central PP_*Mean*_ in N3; frontal and central Upstate% in NREM; and frontal Upstate% in N2 and N3) with derivation-specific spindle density and SO number as covariates. All other associations remained statistically significant (*p*-values < 0.036), but PP_*Mean*_ in N3 sleep was degraded to a trend (*t* = 2.048, *p* = 0.053).

In contrast, ΔWake did not associate significantly with SO–spindle%, PP_*Mean*_, or Upstate% in any sleep stage (*p*-values ≥ 0.110). To assess the sleep specificity of the observed SO–spindle-coupling in memory retention, we ran a mixed ANOVA with ΔSleep and ΔWake as within-subject variables. The delay condition did not interact with either PP_*Mean*_ or Upstate% (*p*-values ≥ 0.135).

### Inter-Spindle Synchrony

Investigating how the synchrony of simultaneous sleep spindles related to retention performance included (1) the probability of a seed spindle being overlapped by spindle(s) in other channels (PD) and (2) the degree of oscillation synchrony (i.e., PLI) between the co-occurring spindles. The mean overlap time across all seed–trailing spindle pairs was 1.003 s. First, we tested whether the channel-pair-wise grand mean raw PLIs differed from the surrogate values and found that they differed significantly (*p*-values < 0.001, Bonferroni-corrected), indicating that simultaneous spindles show higher PLI than randomly paired spindles.

**FIGURE 5:**
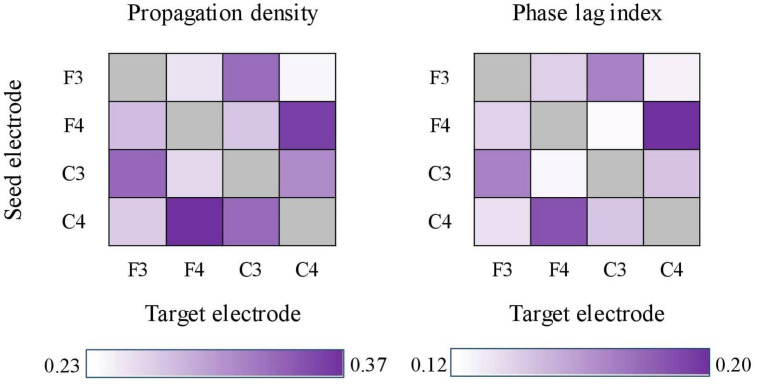
Heat-maps showing propagation density (left) and corrected phase lag indices (cPLIs) between electrode pairs. Both heat-maps show a tendency for bi-directionally higher values between electrodes located in the anterior–posterior axis (e.g., F4–C4 and C4–F4).

For both PD and cPLI, we next examined whether the grand means across the channel pairs differed. Friedman’s test (due to unequal variances) showed statistically significant differences in PD (χ^2^ = 113.369, *p* < 0.001) and cPLI (χ^2^ = 47.825, *p* < 0.001). PD ranged between 0.237 (F3–C4) and 0.372 (C4–F4), and cPLI ranged between 0.122 (F3–C4) and 0.198 (F3–C3). The inter-spindle measures are heat-mapped in Figure 5. The channel pair grand means correlated statistically significantly between PD and cPLI (*r* = 0.773, *p* = 0.003), indicating that the probability of propagation between certain electrodes is associated with phase synchrony between electrodes. In Figure 5, both PD and cPLI appear higher between electrodes in the anterior–posterior axis.

In relation to memory retention, we examined PD and cPLI within four mean variables: frontal to central (F-to-C), C-to-F, left-to-right, and right-to-left. With regard to PD, no statistically significant associations were found with ΔSleep or ΔWake (*p*-values ≥ 0.089). The level of corrected PLIs (cPLIs) between co-occurring spindles did not show statistically significant associations with retention over sleep (*p*-values ≥ 0.150) or waking conditions (*p*-values ≥ 0.080).

## Discussion

We examined how the learning of novel figurative associations (that is, non-conventional metaphors; Herkman and Service, 2008) was associated with the interplay between SOs and sleep spindles, as well as with the synchronization dynamics of overlapping spindles. Furthermore, experimentally coupling respiratory phase and metaphor encoding enabled us to investigate the possibly diverging impact of inhalation and exhalation on memory performance. We observed a strong association between the timing of SO–spindle coupling and overnight memory retention. However, inter-spindle synchronization or respiratory phase was not associated with learning outcomes in the current study.

In our study, fast sleep spindles showed a preferential occurrence close to the positive SO peak. The degree of this coupling predicted memory retention, such that the tendency for spindles to peak close to the SO upstate was associated with less forgetting. This investigation contributes to the convergent evidence observed within behavioral studies (Helfrich et al., 2018; Mikutta et al., 2019; Muehlroth et al., 2019; Hahn et al., 2020). Notably, we showed that memory benefit depended on event-related coupling between prominent SO and spindle events, instead of non-specific modulation between their respective frequency ranges (that is, the MI; Tort et al., 2010) over NREM sleep. This pattern replicates previous findings (Mikutta et al., 2019), wherein the retention of words was associated with the mean PP of sigma power in SO events, but not with MI. Overnight retention was most robustly predicted by the exact timing of spindles in the SO upstate. This finding was most evident when examining NREM sleep as a whole, although separate analyses restricted to N2 and N3 stages showed that SO-upstate-coupled spindles promote memory consolidation regardless of the NREM stage. The significance of SO upstate in spindle-related memory consolidation is presumably related to a specific sequence of excitatory and inhibitory inputs during SO-upstate-nested spindles, exhibiting markedly higher neuronal calcium activity in comparison with isolated SO or spindle events (Niethard et al., 2018). Such a state of optimal synaptic plasticity is not explicitly reflected in MI, which is not tied to specific events. It should be noted that the probability of N3 spindles peaking during an SO cycle, regardless of its phase, also correlated with better retention. Spindles during N3 peak more regularly in the SO upstate, compared with N2 spindles, and presumably the mere amount of SO-upstate spindles in N3 sleep explains this finding.

Interaction tests between the delay conditions (sleep/wake) and the PP variables did not indicate sleep specificity on the memory outcome. One possibility for these findings is that coupling accuracy expresses trait-like learning ability. Previous reports suggest that spindle properties are related to immediate learning cognitive abilities (Schabus et al., 2006), suggesting efficient thalamocortical circuitry (Schabus et al., 2006; Fogel and Smith, 2011; Lustenberger et al., 2012). Although there is currently no research on how SO–spindle coupling is related to cognitive ability, there is evidence that gray matter integrity in medial frontal areas does indeed affect synchrony (Helfrich et al., 2018), potentially reflecting trait-like cognitive capability (Ohtani et al., 2014). Another reason for the non-significant difference between the delay conditions may be the specific properties of our study. For example, some participants in our limited sample approached the ceiling in sleep-containing retention, narrowing the variance of our examined variables and thus limiting our resolution of sleep specificity.

In contrast to SO–spindle coupling, we did not observe any correlation between overnight memory retention and inter-spindle synchronization dynamics. First, the probability of a novel spindle event being temporally overlapped by another spindle in a different channel, combined with the directional preference of such a sequence (or propagation), was not associated with any of the memory outcome measures. Provided that these overlapping spindles represent “matrix” spindles, our findings do not support their hypothesized role in consolidating widespread memory representations (Piantoni et al., 2016). However, simultaneous EEG-recorded spindle events do not necessarily represent only matrix spindles. Core spindles, *via* corticothalamic modulation, can trigger the matrix pathway within a few oscillatory cycles (Piantoni et al., 2016). Hence, any learning-dependent increase of specifically matrix spindles would be obscured by diffused spindle activity triggered by a seed core spindle.

Acknowledging our inability to fractionate the thalamic origins of EEG spindle events, we focused on the degree of cortical synchronization. To this end, we examined the PLI (Stam et al., 2007) between simultaneous spindles. Neural oscillatory synchrony between distinct brain areas is essential for executive functioning (Mizuhara and Yamaguchi, 2007; Sadaghiani et al., 2012; Cavanagh and Frank, 2014) and memory performance over short delays (Schack and Weiss, 2005; Palva et al., 2010; Rutishauser et al., 2010). In our study, simultaneous fast spindles were implicated with intra-hemispherically emphasized phase synchrony, similar to previous findings (Zerouali et al., 2014). We did not find such synchrony to predict memory outcomes. Research on phase synchrony and long-term memory has focused on the times of encoding and retrieval, processes modulated by theta and gamma oscillations (Jutras and Buffalo, 2010; Fell and Axmacher, 2011). The nature of sleep spindles as facilitators of local plasticity (Genzel et al., 2014) raises the question of whether their globalization serves any activity-dependent need or, rather, reflects the cortical state and cortico-thalamocortical properties (Fernandez and Lüthi, 2020). However, it should be noted that the coverage of our EEG setup was limited. Although frontal areas have been reported as a prominent source of propagated spindles, further efforts should increase the resolution to cover temporal spindles (Piantoni et al., 2017).

This was the first study to experimentally test the effect of respiratory phase on memory encoding. We did not observe any impact of respiratory phase at the encoding phase on memory outcomes after a short or long delay, regardless of the delay condition. This was unlikely due to any attentional demands caused by consciously pacing the breathing; instead, volitional control and pacing of breathing may increase coherence in several brain areas, including the frontal and temporal cortices (Herrero et al., 2018). Moreover, a study on respiratory modulation of memory showed (with a control task) that attending breathing caused no differences in memory accuracy (Zelano et al., 2016). However, in that study, nasal inhalation increased the accuracy of visual object recognition. It is noteworthy that this benefit was more strongly related to the moment of retrieval than to encoding. While the subsample in the experimental condition of nasal-only respiration was small (*n* = 11), warranting replication with a larger sample, it is possible that using paced respiration only during encoding (as in our study) does not evoke statistically significant effects. It is of note that prior evidence on the respiratory benefits on memory (Zelano et al., 2016; Arshamian et al., 2018) is based on a recognition memory task, in contrast to our cued recall. Other studies have found that prestimulus hippocampal theta power (a frequency range modulated by respiration; Zelano et al., 2016) during encoding affects recognition but not recall (Merkow et al., 2014). Further research is needed to unveil the exact properties and potential of respiration in memory performance.

The current study also contributes to the literature by introducing more complex linguistic learning materials in relation to sleep than previously implemented. No previous study on sleep and memory has deployed novel metaphor tasks as the learning material, although they seem to yield an ecologically relevant evaluation of linguistic and semantic functioning. We found that the normative difficulty level of the novel metaphors, validated previously (Herkman and Service, 2008), affected the probability of successful recall. This association remained convergent regardless of the type of delay (sleep/wake). Hence, this study added to the understanding that sleep does not differ from waking in “preferring” the long-delay maintenance of difficult metaphorical associations over more easily processed ones. Converging studies from word–pair retention tasks comparing semantically related and unrelated associations have been mixed. One study found an interaction between 12-h delay conditions (sleep/wake) and semantic relatedness (Payne et al., 2012), whereas another report (Lo et al., 2014) displayed a pattern resembling our convergent correlations. It is possible that the novelty in the metaphoric associations and the equalized memory strategy in our task (i.e., to form a mental image of the metaphors, instead of merely instructing participants to “learn” them, as in the studies above) contributed to minimizing the dependence on previous knowledge with respect to the memorized items. This could presumably even their sleep dependency (Schmidt et al., 2006), making the difference between wake and sleep constant.

### Strengths and Limitations

In this study, we replicated previous observations that the coupling accuracy between SOs and fast spindles is essential for verbal memory retention during sleep. While this finding indicates the sensitivity of our memory task to sleep-related mechanisms, it also substantiates the insignificance of inter-spindle synchrony on memory retention. Indeed, no previous study has addressed whether the parameters of simultaneous/propagated spindles relate to memory performance and our results work as an opening in the topic. Finally, by combining metaphor encoding with the respiratory phase, we were able to assess the assumedly improved potentiation of inhalation-tied items. However, no such benefit was observed, which contributed to the delineation of the phenomenon.

There are several limitations that need to be considered. First, the findings on sleep-related mechanisms and memory outcomes are correlational, disallowing any causal interpretations. Second, our EEG montage was limited in terms of thoroughly investigating spindle propagation characteristics. While we covered the fronto-central areas that have been repeatedly associated with verbal memory (Clemens et al., 2005; Mikutta et al., 2019; Muehlroth et al., 2019; Hahn et al., 2020), the temporal lobe has been reported as a prominent source of propagated spindles (Piantoni et al., 2017). Hence, conducting wider-scale EEG would provide more representative results. Third, our metaphor task may have been too easy to provide full variance in the scrutinized variables. The recall rates in the immediate and sleep conditions were at or close to the maximum scores, limiting the variance of retention measures. Lastly, besides the instruction and visual cues, we did not exert other control measures and we did not evaluate the participants’ respiration. Therefore, we cannot verify the exact phase-item match during encoding, and the use of a pneumotachometer in further studies is thus recommended.

## Conclusion

The overnight retention of metaphors is associated with accurate coupling between SO upstate and sleep spindles. On the other hand, our novel aim to examine the temporal and phasic interplay between co-occurring NREM spindles did not reveal any influence on memory outcomes. The phase of nasal respiration during encoding did not affect memory performance, warranting questions on whether inhalation-specific benefits are more evidently tied to other memory processes (consolidation and retrieval) or types of memorized material (recognition). Our results substantiate the accumulating experimental data that highlights the importance of SO–spindle coupling in memory retention.

## Data Availability Statement

The raw data supporting the conclusions of this article will be made available by the authors on request, without undue reservation.

## Ethics Statement

The studies involving human participants were reviewed and approved by Helsinki and Uusimaa hospital district ethics committee. The patients/participants provided their written informed consent to participate in this study.

## Author Contributions

RH: conceptualization, methodology, writing—original draft preparation, formal analysis, investigation, and visualization. LK: conceptualization, review, and editing. MA: data collection, review, and editing. A-KP: conceptualization, writing—review and editing, supervision, and project administration. All authors contributed to the article and approved the submitted version.

## Conflict of Interest

The authors declare that the research was conducted in the absence of any commercial or financial relationships that could be construed as a potential conflict of interest.

## Publisher’s Note

All claims expressed in this article are solely those of the authors and do not necessarily represent those of their affiliated organizations, or those of the publisher, the editors and the reviewers. Any product that may be evaluated in this article, or claim that may be made by its manufacturer, is not guaranteed or endorsed by the publisher.

## Footnotes

1
https://clinicaltrials.gov/ct2/show/NCT02964598

2
https://github.com/wonambi-python/wonambi

## References

[B1] Alger S. E. Chen S. Payne J. D. (2019). Do different salience cues compete for dominance in memory over a daytime nap? *Neurobiol. Learn. Mem.* 160 48–57. 10.1016/j.nlm.2018.06.005 29906574 PMC6291383

[B2] Arshamian A. Iravani B. Majid A. Lundström J. N. (2018). Respiration modulates olfactory memory consolidation in humans. *J. Neurosci.* 38 10286–10294. 10.1523/jneurosci.3360-17.2018 30348674 PMC6596216

[B3] Arzouan Y. Goldstein A. Faust M. (2007). Dynamics of hemispheric activity during metaphor comprehension: electrophysiological measures. *Neuroimage* 36 222–231. 10.1016/j.neuroimage.2007.02.015 17428685

[B4] Bahramisharif A. Van Gerven M.a.J Aarnoutse E. J. Mercier M. R. Schwartz T. H. Foxe J. J. (2013). Propagating neocortical gamma bursts are coordinated by traveling alpha waves. *J. Neurosci.* 33 18849–18854. 10.1523/jneurosci.2455-13.2013 24285891 PMC4262700

[B5] Bambini V. Gentili C. Ricciardi E. Bertinetto P. M. Pietrini P. (2011). Decomposing metaphor processing at the cognitive and neural level through functional magnetic resonance imaging. *Brain Res. Bull.* 86 203–216. 10.1016/j.brainresbull.2011.07.015 21803125

[B6] Beck A. T. Steer R. A. Brown G. K. (1996). *Manual for the Beck Depression Inventory-II.* San Antonio, TX: Psychological Corporation.

[B7] Benchenane K. Peyrache A. Khamassi M. Tierney P. L. Gioanni Y. Battaglia F. P. (2010). Coherent theta oscillations and reorganization of spike timing in the hippocampal- prefrontal network upon learning. *Neuron* 66 921–936. 10.1016/j.neuron.2010.05.013 20620877

[B8] Bennion K. A. Payne J. D. Kensinger E. A. (2016). The impact of napping on memory for future-relevant stimuli: prioritization among multiple salience cues. *Behav. Neurosci.* 130 281–289. 10.1037/bne0000142 27214500

[B9] Berens P. (2009). CircStat: a matlab toolbox for circular statistics. *J. Statist. Softw.* 31 1–21.

[B10] Bohrn I. C. Altmann U. Jacobs A. M. (2012). Looking at the brains behind figurative language–a quantitative meta-analysis of neuroimaging studies on metaphor, idiom, and irony processing. *Neuropsychologia* 50 2669–2683. 10.1016/j.neuropsychologia.2012.07.021 22824234

[B11] Bonjean M. Baker T. Bazhenov M. Cash S. Halgren E. Sejnowski T. (2012). Interactions between core and matrix thalamocortical projections in human sleep spindle synchronization. *J. Neurosci.* 32 5250–5263. 10.1523/jneurosci.6141-11.2012 22496571 PMC3342310

[B12] Cavanagh J. F. Frank M. J. (2014). Frontal theta as a mechanism for cognitive control. *Trends Cogn. Sci.* 18 414–421. 10.1016/j.tics.2014.04.012 24835663 PMC4112145

[B13] Chauvette S. Seigneur J. Timofeev I. (2012). Sleep oscillations in the thalamocortical system induce long-term neuronal plasticity. *Neuron* 75 1105–1113. 10.1016/j.neuron.2012.08.034 22998877 PMC3458311

[B14] Clemens Z. Fabó D. Halász P. (2005). Overnight verbal memory retention correlates with the number of sleep spindles. *Neuroscience* 132 529–535. 10.1016/j.neuroscience.2005.01.011 15802203

[B15] Cohen N. Pell L. Edelson M. G. Ben-Yakov A. Pine A. Dudai Y. (2015). Peri-encoding predictors of memory encoding and consolidation. *Neurosci. Biobehav. Rev.* 50 128–142. 10.1016/j.neubiorev.2014.11.002 25446944

[B16] Combrisson E. Nest T. Brovelli A. Ince R.a.A Soto J. L. P. Guillot A. (2020). Tensorpac: an open-source python toolbox for tensor-based phase-amplitude coupling measurement in electrophysiological brain signals. *PLoS Comput. Biol.* 16:e1008302. 10.1371/journal.pcbi.1008302 33119593 PMC7654762

[B17] Cox R. Mylonas D. S. Manoach D. S. Stickgold R. (2018). Large-scale structure and individual fingerprints of locally coupled sleep oscillations. *Sleep* 41:zsy175. 10.1093/sleep/zsy175 PMC6289240 30184179

[B18] Davidson P. Jönsson P. Carlsson I. Pace-Schott E. (2020). Does sleep selectively strengthen certain memories over others? A critical review of the literature. *PsyArXiv* [Preprint]. 10.2147/NSS.S286701 34335065 PMC8318217

[B19] Delorme A. Makeig S. (2004). EEGLAB: an open source toolbox for analysis of single-trial EEG dynamics including independent component analysis. *J. Neurosci. Methods* 134 9–21. 15102499 10.1016/j.jneumeth.2003.10.009

[B20] Diaz M. T. Eppes A. (2018). Factors influencing right hemisphere engagement during metaphor comprehension. *Front. Psychol.* 9:414. 10.3389/fpsyg.2018.00414 29643825 PMC5883147

[B21] Fell J. Axmacher N. (2011). The role of phase synchronization in memory processes. *Nat. Rev. Neurosci.* 12 105–118. 10.1038/nrn2979 21248789

[B22] Fernandez L. M. J. Lüthi A. (2020). Sleep spindles: mechanisms and functions. *Physiol. Rev.* 100 805–868. 10.1152/physrev.00042.2018 31804897

[B23] Ferrarelli F. Huber R. Peterson M. J. Massimini M. Murphy M. Riedner B. A. (2007). Reduced sleep spindle activity in schizophrenia patients. *Am. J. Psychiatry* 164 483–492. 10.1176/ajp.2007.164.3.483 17329474

[B24] Fogel S. M. Smith C. T. (2011). The function of the sleep spindle: a physiological index of intelligence and a mechanism for sleep-dependent memory consolidation. *Neurosci. Biobehav. Rev.* 35 1154–1165. 10.1016/j.neubiorev.2010.12.003 21167865

[B25] Frauscher B. Bernasconi N. Caldairou B. Von Ellenrieder N. Bernasconi A. Gotman J. (2015). Interictal hippocampal spiking influences the occurrence of hippocampal sleep spindles. *Sleep* 38 1927–1933. 10.5665/sleep.5242 26194569 PMC4667386

[B26] Gais S. Mölle M. Helms K. Born J. (2002). Learning-dependent increases in sleep spindle density. *J. Neurosci.* 22:6830. 10.1523/jneurosci.22-15-06830.2002 12151563 PMC6758170

[B27] Genzel L. Kroes M. C. W. Dresler M. Battaglia F. P. (2014). Light sleep versus slow wave sleep in memory consolidation: a question of global versus local processes? *Trends Neurosci.* 37 10–19. 10.1016/j.tins.2013.10.002 24210928

[B28] Goldstein A. N. Walker M. P. (2014). The role of sleep in emotional brain function. *Annu. Rev. Clin. Psychol.* 10 679–708. 10.1146/annurev-clinpsy-032813-153716 24499013 PMC4286245

[B29] Hahn M. A. Heib D. Schabus M. Hoedlmoser K. Helfrich R. F. (2020). Slow oscillation-spindle coupling predicts enhanced memory formation from childhood to adolescence. *ELife* 9:e53730. 10.7554/eLife.53730 PMC7314542 32579108

[B30] Halonen R. Kuula L. Lahti J. Makkonen T. Räikkönen K. Pesonen A.-K. (2019). BDNF Val66Met polymorphism moderates the association between sleep spindles and overnight visual recognition. *Behav. Brain Res.* 375:112157. 10.1016/j.bbr.2019.112157 31437468

[B31] Helfrich R. F. Mander B. A. Jagust W. J. Knight R. T. Walker M. P. (2018). Old brains come uncoupled in sleep: slow wave-spindle synchrony, brain atrophy, and forgetting. *Neuron* 97 221.e–230.e. 29249289 10.1016/j.neuron.2017.11.020 PMC5754239

[B32] Herkman J. Service E. (2008). Psykologisten dimensioiden normatiiviset arviot 125 metaforasta. *Puhe Ja Kieli* 28 187–206.

[B33] Hermans E. J. Battaglia F. P. Atsak P. De Voogd L. D. Fernández G. Roozendaal B. (2014). How the amygdala affects emotional memory by altering brain network properties. *Neurobiol. Learn. Mem.* 112 2–16. 10.1016/j.nlm.2014.02.005 24583373

[B34] Herrero J. L. Khuvis S. Yeagle E. Cerf M. Mehta A. D. (2018). Breathing above the brain stem: volitional control and attentional modulation in humans. *J. Neurophysiol.* 119 145–159. 10.1152/jn.00551.2017 28954895 PMC5866472

[B35] Jutras M. J. Buffalo E. A. (2010). Synchronous neural activity and memory formation. *Curr. Opin. Neurobiol.* 20 150–155. 10.1016/j.conb.2010.02.006 20303255 PMC2862842

[B36] Klinzing J. G. Niethard N. Born J. (2019). Mechanisms of systems memory consolidation during sleep. *Nat. Neurosci.* 22 1598–1610. 10.1038/s41593-019-0467-3 31451802

[B37] Lai V. T. Van Dam W. Conant L. L. Binder J. R. Desai R. H. (2015). Familiarity differentially affects right hemisphere contributions to processing metaphors and literals. *Front. Hum. Neurosci.* 9:44. 10.3389/fnhum.2015.00044 25713522 PMC4322727

[B38] Liu Y. Mcafee S. S. Heck D. H. (2017). Hippocampal sharp-wave ripples in awake mice are entrained by respiration. *Sci. Rep.* 7:8950. 10.1038/s41598-017-09511-8 PMC5566471 28827599

[B39] Lo J. C. Dijk D.-J. Groeger J. A. (2014). Comparing the effects of nocturnal sleep and daytime napping on declarative memory consolidation. *PLoS One* 9:e108100. 10.1371/journal.pone.0108100 25229457 PMC4168137

[B40] Lustenberger C. Maric A. Dürr R. Achermann P. Huber R. (2012). Triangular relationship between sleep spindle activity. general cognitive ability and the efficiency of declarative learning. *PLoS One* 7:e49561. 10.1371/journal.pone.0049561 23185361 PMC3504114

[B41] Mashal N. Faust M. (2009). Conventionalisation of novel metaphors: a shift in hemispheric asymmetry. *Laterality* 14 573–589. 10.1080/13576500902734645 19253086

[B42] Mednick S. C. Mcdevitt E. A. Walsh J. K. Wamsley E. Paulus M. Kanady J. C. (2013). The critical role of sleep spindles in hippocampal-dependent memory: a pharmacology study. *J. Neurosci.* 33 4494–4504. 10.1523/jneurosci.3127-12.2013 23467365 PMC3744388

[B43] Merkow M. B. Burke J. F. Stein J. M. Kahana M. J. (2014). Prestimulus theta in the human hippocampus predicts subsequent recognition but not recall. *Hippocampus* 24 1562–1569. 10.1002/hipo.22335 25074395 PMC4288746

[B44] Mikutta C. Feige B. Maier J. G. Hertenstein E. Holz J. Riemann D. (2019). Phase-amplitude coupling of sleep slow oscillatory and spindle activity correlates with overnight memory consolidation. *J. Sleep Res.* 28:e12835. 10.1111/jsr.12835 30848042

[B45] Mizuhara H. Yamaguchi Y. (2007). Human cortical circuits for central executive function emerge by theta phase synchronization. *Neuroimage* 36 232–244. 10.1016/j.neuroimage.2007.02.026 17433880

[B46] Muehlroth B. E. Sander M. C. Fandakova Y. Grandy T. H. Rasch B. Shing Y. L. (2019). Precise slow oscillation–spindle coupling promotes memory consolidation in younger and older adults. *Sci. Rep.* 9:1940. 10.1038/s41598-018-36557-z PMC6374430 30760741

[B47] Ngo H. V. Miedema A. Faude I. Martinetz T. Mölle M. Born J. (2015). Driving sleep slow oscillations by auditory closed-loop stimulation-a self-limiting process. *J. Neurosci.* 35 6630–6638. 10.1523/jneurosci.3133-14.2015 25926443 PMC4412888

[B48] Ngo H.-V. Fell J. Staresina B. (2020). Sleep spindles mediate hippocampal-neocortical coupling during long-duration ripples. *ELife* 9:e57011. 10.7554/eLife.57011 PMC7363445 32657268

[B49] Niethard N. Ngo H. -V. V. Ehrlich I. Born J. (2018). Cortical circuit activity underlying sleep slow oscillations and spindles. *Proc. Natl. Acad. Sci. U.S.A.* 39 E9220–E9229. 10.1073/pnas.1805517115 30209214 PMC6166829

[B50] Ohtani T. Nestor P. G. Bouix S. Saito Y. Hosokawa T. Kubicki M. (2014). Medial frontal white and gray matter contributions to general intelligence. *PLoS One* 9:e112691. 10.1371/journal.pone.0112691 25551572 PMC4281236

[B51] O’Reilly C. Nielsen T. (2014). Assessing EEG sleep spindle propagation. Part 2: experimental characterization. *J. Neurosci. Methods* 221 215–227. 10.1016/j.jneumeth.2013.08.014 23999173

[B52] Palva J. M. Monto S. Kulashekhar S. Palva S. (2010). Neuronal synchrony reveals working memory networks and predicts individual memory capacity. *Proc. Natl. Acad. Sci. U.S.A.* 107 7580–7585. 10.1073/pnas.0913113107 20368447 PMC2867688

[B53] Payne J. D. Tucker M. A. Ellenbogen J. M. Wamsley E. J. Walker M. P. Schacter D. L. (2012). Memory for semantically related and unrelated declarative information: the benefit of sleep, the cost of wake. *PLoS One* 7:e33079. 10.1371/journal.pone.0033079 22457736 PMC3310860

[B54] Peyrache A. Seibt J. (2020). A mechanism for learning with sleep spindles. *Philos. Trans. R. Soc. Lond. B Biol. Sci.* 375:20190230. 10.1098/rstb.2019.0230 32248788 PMC7209910

[B55] Peyrache A. Khamassi M. Benchenane K. Wiener S. I. Battaglia F. P. (2009). Replay of rule-learning related neural patterns in the prefrontal cortex during sleep. *Nat. Neurosci.* 12 919–926. 10.1038/nn.2337 19483687

[B56] Piantoni G. Halgren E. Cash S. S. (2016). The contribution of thalamocortical core and matrix pathways to sleep spindles. *Neural Plast.* 2016:3024342. 10.1155/2016/3024342 PMC4842069 27144033

[B57] Piantoni G. Halgren E. Cash S. S. (2017). Spatiotemporal characteristics of sleep spindles depend on cortical location. *Neuroimage* 146 236–245. 10.1016/j.neuroimage.2016.11.010 27840241 PMC5321858

[B58] Piantoni G. O’Byrne J. (2021). *Wonambi: EEG Analysis Toolbox Version 6.13*. Available online at: https://github.com/wonambi-python/wonambi

[B59] Rasch B. Born J. (2013). About sleep’s role in memory. *Physiol. Rev.* 93 681–766. 23589831 10.1152/physrev.00032.2012 PMC3768102

[B60] Rutishauser U. Ross I. B. Mamelak A. N. Schuman E. M. (2010). Human memory strength is predicted by theta-frequency phase-locking of single neurons. *Nature* 464 903–907. 10.1038/nature08860 20336071

[B61] Sadaghiani S. Scheeringa R. Lehongre K. Morillon B. Giraud A.-L. D’esposito M. (2012). Alpha-band phase synchrony is related to activity in the fronto-parietal adaptive control network. *J. Neurosci.* 32 14305–14310. 10.1523/jneurosci.1358-12.2012 23055501 PMC4057938

[B62] Schabus M. Hodlmoser K. Gruber G. Sauter C. Anderer P. Klosch G. (2006). Sleep spindle-related activity in the human EEG and its relation to general cognitive and learning abilities. *Eur. J. Neurosci.* 23 1738–1746. 10.1111/j.1460-9568.2006.04694.x 16623830

[B63] Schack B. Weiss S. (2005). Quantification of phase synchronization phenomena and their importance for verbal memory processes. *Biol. Cybern.* 92 275–287. 10.1007/s00422-005-0555-1 15818488

[B64] Schmidt C. Peigneux P. Muto V. Schenkel M. Knoblauch V. Munch M. (2006). Encoding difficulty promotes postlearning changes in sleep spindle activity during napping. *J. Neurosci.* 26 8976–8982. 10.1523/jneurosci.2464-06.2006 16943553 PMC6675334

[B65] Souza R. T. F. D. Gerhardt G. J. L. Schönwald S. V. Rybarczyk-Filho J. L. Lemke N. (2016). Synchronization and propagation of global sleep spindles. *PLoS One* 11:e0151369. 10.1371/journal.pone.0151369 26963102 PMC4786112

[B66] Stam C. J. Nolte G. Daffertshofer A. (2007). Phase lag index: assessment of functional connectivity from multi channel EEG and MEG with diminished bias from common sources. *Hum. Brain Mapp.* 28 1178–1193. 10.1002/hbm.20346 17266107 PMC6871367

[B67] Steriade M. (2006). Grouping of brain rhythms in corticothalamic systems. *Neuroscience* 137 1087–1106. 10.1016/j.neuroscience.2005.10.029 16343791

[B68] Studte S. Bridger E. Mecklinger A. (2017). Sleep spindles during a nap correlate with post sleep memory performance for highly rewarded word-pairs. *Brain Lang.* 167 28–35. 10.1016/j.bandl.2016.03.003 27129616

[B69] Tort A. B. L. Komorowski R. Eichenbaum H. Kopell N. (2010). Measuring phase-amplitude coupling between neuronal oscillations of different frequencies. *J. Neurophysiol.* 104 1195–1210. 10.1152/jn.00106.2010 20463205 PMC2941206

[B70] Williams N. (2014). The GAD-7 questionnaire. *Occup. Med.* 64 224–224. 10.1093/occmed/kqt161

[B71] Zelano C. Jiang H. Zhou G. Arora N. Schuele S. Rosenow J. (2016). Nasal respiration entrains human limbic oscillations and modulates cognitive function. *J. Neurosci.* 36 12448–12467. 27927961 10.1523/JNEUROSCI.2586-16.2016 PMC5148230

[B72] Zerouali Y. Lina J. M. Sekerovic Z. Godbout J. Dube J. Jolicoeur P. (2014). A time-frequency analysis of the dynamics of cortical networks of sleep spindles from MEG-EEG recordings. *Front. Neurosci.* 8:310. 10.3389/fnins.2014.00310 25389381 PMC4211563

