# Supplementary material for: The Overnight Retention of Novel Metaphors Associates With Slow Oscillation–Spindle Coupling but Not With Respiratory Phase at Encoding
Source: Front Behav Neurosci. 2021 Aug 31;15:712774. doi: 10.3389/fnbeh.2021.712774 (PMC8439423; doi:10.3389/fnbeh.2021.712774)
Supplement: Supplementary file 1 [file Data_Sheet_1.docx]

| Table S1. The regression between SO-spindle coupling and overnight retention in NREM sleep | | | | | | | |  |  |
| --- | --- | --- | --- | --- | --- | --- | --- | --- | --- |
|  | Sex partialled out | | | |  | Control Model | | | |
|  | PP_Mean_ | | Upstate% | |  | PP_Mean_ | | Upstate% | |
| Channel | t | p | t | p |  | t | p | t | p |
| F | 2.508* | .019 | -3.796*** | <.001 |  | 2.560* | .019 | -3.486** | .002 |
| F4 | 2.085* | .048 | -3.492** | .002 |  | 2.133* | .046 | -3.009** | .007 |
| F3 | 2.382* | .026 | -3.597** | .001 |  | 2.362* | .028 | -3.457** | .002 |
| C | 1.532 | .139 | -2.880** | .008 |  | 1.498 | .150 | -2.531* | .019 |
| C4 | 1.259 | .220 | -2.825** | .009 |  | 1.314 | .204 | -2.430* | .024 |
| C3 | 1.014 | .321 | -2.111* | .045 |  | 0.983 | .337 | -1.934 | .064 |
| Control Model = covariates include sleep duration, age, BDI score and GAD-7 score. SO = slow oscillation. F = Frontal. C = Central. PPMean = the preferred phase of spindle peak amplitude in slow oscillation cycle. Upstate% = the percentage of SO-spindles peaking +- 30 degrees from 0 degrees.t = t-value in regression analysis. p = p-value. ***: p value < .001; **: p value < .01. *: p value < .05. | | | | | | | | | |

|  | Table S2. The regression between SO-spindle coupling and overnight retention in N2 and N3 sleep. | | | | | | | | |  |  |
| --- | --- | --- | --- | --- | --- | --- | --- | --- | --- | --- | --- |
|  | |  | Sex partialled out | | | |  | Control Model | | | |
|  | |  | PP_Mean_ | | Upstate% | |  | PP_Mean_ | | Upstate% | |
| Channel | | Stage | t | p | t | p |  | t | p | t | p |
| F | | N2 | -0.007 | .994 | -2.368* | .026 |  | 0.625 | .539 | -2.107* | .046 |
| C | | N2 | 0.571 | .573 | -1.712 | .099 |  | 0.929 | .364 | -1.311 | .204 |
| F | | N3 | 1.693 | .103 | -3.280** | .003 |  | 1.446 | .164 | -2.616* | .016 |
| C | | N3 | 2.372* | .026 | -1.869 | .073 |  | 2.227* | .038 | -1.486 | .152 |
|  | Control Model = covariates include sleep duration, age, BDI score and GAD-7 score. SO = slow oscillation. F = Frontal. C = Central. PPMean = the preferred phase of spindle peak amplitude in slow oscillation cycle. Upstate% = the percentage of SO-spindles peaking +- 30 degrees from 0 degrees.t = t-value in regression analysis. p = p-value. **: p-value < .01. *: p-value < .05. | | | | | | | | | | |
